# Case report: Remarkable response to a novel combination of mitotane, etoposide, paraplatin, and sintilimab in a patient with metastatic adrenocortical carcinoma

**DOI:** 10.3389/fendo.2023.1115893

**Published:** 2023-09-08

**Authors:** Yan Weng, Lin Wang, Xiao-Yi Wang, Xin-Xiang Fan, Li Yan, Zhi-Hua Li, Shao-Ling Zhang

**Affiliations:** ^1^ Department of Endocrinology, Sun Yat-sen Memorial Hospital, Sun Yat-sen University, Guangzhou, China; ^2^ Department of Pathology, Sun Yat-sen Memorial Hospital, Sun Yat-sen University, Guangzhou, China; ^3^ Department of Urology, Sun Yat-sen Memorial Hospital, Sun Yat-sen University, Guangzhou, China; ^4^ Department of Oncology, Sun Yat-sen Memorial Hospital, Sun Yat-sen University, Guangzhou, China

**Keywords:** adrenocortical carcinoma, MSI-H, MLH1, tumor mutational burden, PD-1

## Abstract

**Background:**

Adrenocortical carcinoma (ACC) is a rare malignancy with a poor prognosis and limited treatment options for metastases. However, new effective regimens are emerging for specific conditions in metastatic ACC.

**Case presentation:**

We report a case of a 36-year-old man diagnosed with metastatic ACC who had a large left adrenal mass (158 mm × 112 mm) and multiple metastases in the liver and lungs. Genetic testing revealed a microsatellite instability-high (MSI-H) tumor, a splice mutation in MLH1, and a high tumor mutational burden (TMB). After the left adrenalectomy, he received sequential treatment with a combination of mitotane, etoposide, paraplatin (EP-M), and sintilimab. His condition has been assessed as a stable disease since the sixth cycle of the combined regimen.

**Conclusion:**

This case highlights the remarkable response of our patient’s ACC with MSI-H tumor, MLH1 spice mutation, and high TMB to treatment with a novel combination of EP-M and sintilimab. Our findings suggest a promising therapeutic option for patients with similar molecular profiles.

## Background

Adrenocortical carcinoma (ACC) is a rare malignancy with an incidence of 0.5–2 per million person-years. Although ACC is typically an aggressive tumor, its prognosis is largely dependent on tumor staging (1). The 5-year survival of ACC rapidly decreases with advancing oncology stages: stage I (66% to 82%), stage II (58% to 64%), stage III (24% to 50%), and stage IV (0% to 17%) (2).

Treatment options for advanced ACC are limited. In general, first-line therapy in patients with metastatic ACC is mitotane alone or mitotane plus chemotherapy (3). Surgery may be used to control tumor growth and hypersecretion-related symptoms and prolong survival in selected patients. Mitotane and chemotherapy have limited effectiveness in treating advanced ACC (1). However, new effective regimens are emerging, particularly in cases of ACC with specific gene mutations. Currently, immunotherapy (such as antiprogrammed cell death protein 1 (PD-1)/antiprogrammed cell death ligand-1 (PD-L1) agents) has changed the treatment paradigm for certain cancers, including melanoma, lung cancer, and renal cancer (4). Patients with malignant tumors who carry specific genetic mutations in DNA mismatch repair (MMR) enzymes or have high TMB are expected to have better outcomes with immunotherapy (5, 6).

## Case presentation

We present the case of a 36-year-old man from Hunan Province, China, with severe hypertension and hypokalemia and without any history of glucocorticoid exposure. Clinically, the patient had a 7-year duration of hypertension (160–180/90–110 mmHg) with an elevated body mass index (25.6 kg/m^2^). Physical examination revealed no manifestations of Cushing’s syndrome, such as the moon face and the buffalo neck. No family history of hypertension, endocrine tumors, or Lynch disorder was found.

**Figure 1:**
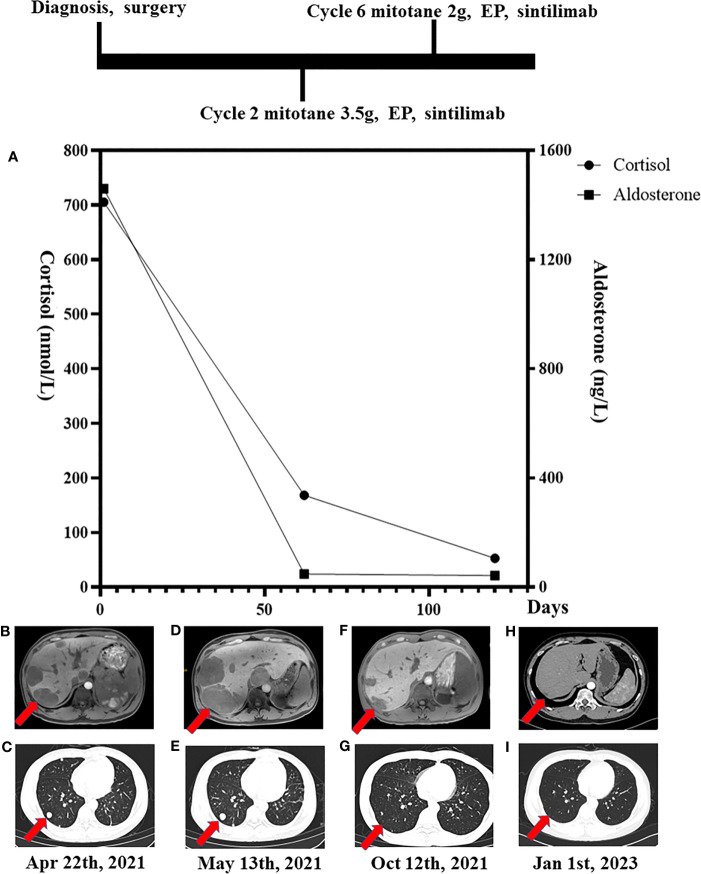
Timeline of laboratory results. **(A)** The cortisol and aldosterone concentrations plotted against time. Magnetic resonance imaging and computerized tomography showed multiple metastases in both the liver **(B, D, F, H)** and lungs **(C, E, G, I)** at diagnosis and following initiation of therapy.

The patient’s blood, urine, stool tests, and renal and liver functions were normal. Biochemical tests showed marked autonomous adrenocorticotrophic hormone-independent hypercortisolemia. Plasma cortisol was 705.21 nmol/L at 8 a.m. and 680.50 nmol/L at 24 p.m. (reference range: 118.60–610.00 nmol/L), plasma adrenocorticotropic hormone (ACTH) was < 5 pg/mL (reference range: 0–46 pg/mL), indicating excessive cortisol and disturbed rhythm (**Figure 1A** **)**. The patient’s late-night salivary cortisol was 53.84 nmol/L (reference range: 0.00–10.40 nmol/L), and his urinary free cortisol was 3,211.2 nmol/24 h (reference range: 153.2–789.4 nmol/24 h). Furthermore, plasma cortisol was not suppressed by 1 mg of dexamethasone (725.77 nmol/L) administered overnight. The patient’s serum potassium was 2.3 mmol/L, plasma renin activity was 0.78 ng/mL/h (reference range: 0.10–6.56 ng/mL/h), and plasma aldosterone concentration was 1,460.0 ng/L (reference range: 70.0–300.0 ng/L). The patient underwent a captopril challenge test; premedication plasma aldosterone concentration was 676.0 ng/L and plasma renin activity was 0.94 ng/mL/h; postmedication plasma aldosterone concentration was 799.0 ng/L and plasma renin activity was 0.61 ng/mL/h. Vanillic amygdalin assay in the urine during a 24-h period was 10.2 mg/24 h (reference range: 0–12.0 mg/24 h), metanephrine was 0.06 nmol/L (reference range: ≤ 0.60 nmol/L), and methoxynorepinephrine was 0.14 nmol/L (reference range: ≤ 0.90 nmol/L). The thyroid function test reported that the level of thyroid-stimulating hormone (TSH) was 2.047 mU/L (reference range: 0.550–4.780 mU/L), free triiodothyronine (FT3) was 5.58 pmol/L (reference range: 3.50–6.50 pmol/L), and free thyroxine (FT4) was 15.03 pmol/L (reference range: 11.50–22.70 pmol/L). His plasma testosterone was 5.67 nmol/L (reference range: 6.07–27.10 nmol/L) and dehydroepiandrosterone-sulfate was 80.88 μg/dL (reference range: 88.90–427.00 μg/dL). An analysis of the patient’s plasma steroid metabolites by liquid chromatography-tandem mass spectrometry revealed an elevated secretion of 11-deoxycorticosterone and 11-deoxycortisol.

**Figure 2:**
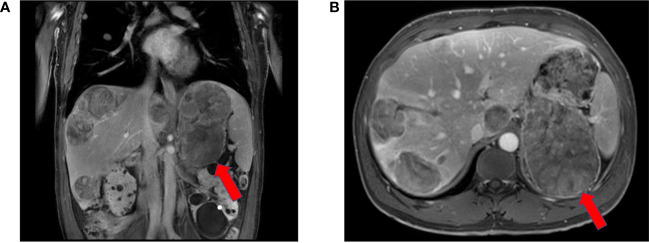
Red arrowheads indicate the primary tumor at diagnosis. Contrast-enhanced magnetic resonance imaging revealed a 158 mm × 112 mm heterogeneously enhanced left suprarenal mass with necrosis.

**Figure 3:**
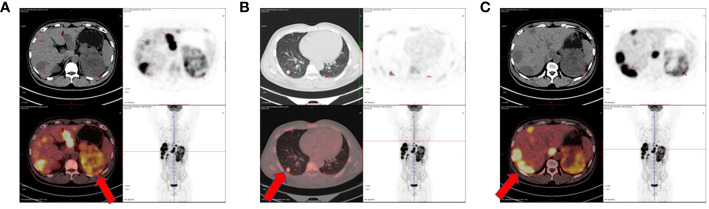
Positron emission tomography-computed tomography showed primary tumor in the left adrenal gland **(A)** and multiple metastases in both lungs **(B)** and liver **(C)** before treatment. The red arrowheads showed the primary lesion of the left adrenal gland and the metastatic lesions of the lungs and liver.

Magnetic resonance imaging (MRI) showed a large left adrenal mass (158 mm × 112 mm) compressing the left kidney, with multiple liver and lung metastases. MRI also showed partially high density on T2-emphasizing phase images and low density on T1-emphasizing phase images **(** **Figure 2** **)**. There were 14 metastases in the liver, and the largest metastasis was about 44 mm in diameter. Multiple metastases were visible in both lungs, with a maximum lesion of about 15 mm **(** **Figures 1B, C** **)**. Fluorodeoxyglucose positron emission tomography showed a strong accumulation in the left adrenal mass (SUV max of 8.8, **Figure 3A**) and metastatic lesions in the lung (SUV max of 8.7, **Figure 3B**) and liver (SUV max of 17.1, **Figure 3C**), but no lesions were detected in the bone or lymph nodes.

**Figure 4:**
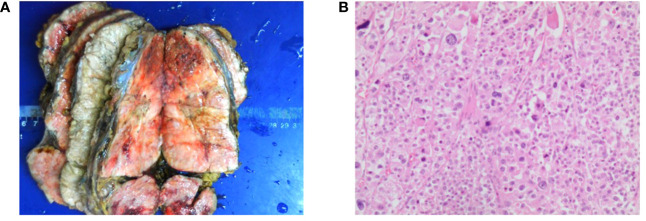
Presentation of the resected adrenocortical carcinoma (ACC) tumor tissue. **(A)** Resected (ACC) tumors. The size of the tumor is 17 cm × 10 cm × 8 cm. **(B)** Representative hematoxylin and eosin-stained photomicrograph of ACC (scale bar = 200 μm).

The patient had such resistant hypertension that five antihypertensives daily were required to maintain blood pressure between 173 and 200/100 and 130 mmHg. After 26 g of potassium chloride supplementation, the serum potassium was corrected to 3.2 mmol/L. When the patient’s general condition improved, he then underwent the palliative left adrenalectomy on 22nd April 2021, and a subsequent tumor biopsy confirmed the diagnosis of ACC (**Figure 4A**), with a Weiss score of 7 points. The pathological results revealed vascular invasion with a Ki-67% of 75% and a mitotic count of 40/50 high-power field (**Figure 4B**). Immunohistochemical results indicated that α-inhibin (+), CD56 (+), Syn (partially+), Melan-A (+), CgA (−), NSE (partially+), and S-100 (−) were present. Thus, the patient was diagnosed with ACC of the left adrenal gland, with multiple metastases in the liver and both lungs (stage IV, T4N0M1).

Additionally, the patient’s genetic test identified an MLH1 nonsense mutation and an NF1 frameshift mutation, along with a synonymous variant in exon 4 of the *TP53* gene. The patient also had a microsatellite instability-high (MSI-H) tumor with a TMB of 19.9 mut/Mb.

After surgery, the patient was initially administered mitotane at 0.5 g/day, which was gradually increased to 4 g/day within a month and then decreased to 1 g/day after combination therapy (**Table 1**). From 8 May 2021 to 3 November 2021, the patient received eight cycles of chemotherapy with etoposide and paraplatin (EP regime: etoposide 200 mg Days 1–3 + paraplatin 500 mg Day 1). After the first cycle of EP-M, he was assessed as having progressive disease (PD): the maximum size of the liver lesions grew to 64 mm and that of the lung lesion grew to 16 mm (**Figures 1D, E** **)**. Thus, the patient began an add-on therapy with programmed death-1 (PD-1) immunotherapy using sintilimab (200 mg once a month) on 1st June 2021. After four cycles of EP-M and sintilimab, the metastatic lesions continued to shrink, and the patient’s condition was assessed as stable disease (SD) at the sixth cycle, the maximum size of the liver lesion decreased to 24 mm and that of the lung lesion decreased to 4 mm (**Figures 1F, G** **)**. Following eight cycles of EP, the patient continued to receive sintilimab and mitotane only, and eventually, sintilimab monotherapy was used to maintain SD (**Table 1**). The computed tomography (CT) scan on 1st January 2023 showed a reduction of ACC metastases both in the liver and lungs. The original maximum liver lesion was approximately 17 mm in diameter and the original maximum lung lesion disappeared. Moreover, neither relapsed disease at the primary site nor new metastases were detected **(** **Figures 1H, I** **)**. On 20th July 2022, his blood pressure was stabilized in a normal range without antihypertensives, and the hypokalemia was corrected without potassium supplements.

**Table 1:** Timeline of the treatment.

Starting date	Treatment	Side effects
22nd April 2021	Left adrenalectomy	
1st May 2021	Adjuvant mitotane at 0.5 g	
8th May 2021	Adjuvant mitotane at 1.5 g, EP	
1st June 2021	Adjuvant mitotane at 4 g, EP, sintilimab	
24th June 2021	Adjuvant mitotane at 3.5 g, EP, sintilimab	
25th July 2021	Adjuvant mitotane at 3 g, EP sintilimab	Hypothyroidism and hypoadrenocorticism
20th August 2021	Adjuvant mitotane at 2 g, EP, sintilimab	
15th September 2021	Adjuvant mitotane at 1 g, EP, sintilimab	
12th October 2021	Adjuvant mitotane at 1 g, EP, sintilimab	
3rd November 2021	Adjuvant mitotane at 1 g, EP, sintilimab	
17th December 2021	Adjuvant mitotane at 1 g, sintilimab	
13th January 2022	Adjuvant mitotane at 1g, sintinimab	
10th February 2022	Adjuvant mitotane at 1 g, sintinimab	
8th March 2022	Adjuvant mitotane at 1 g, sintinimab	
11th April 2022	Adjuvant mitotane at 1 g, sintinimab	
9th May 2022	Adjuvant mitotane at 1 g, sintinimab	
9th June 2022	Adjuvant mitotane at 1 g, sintinimab	
5th July 2022	Adjuvant mitotane at 1 g, sintinimab	
28th July 2022	Adjuvant mitotane at 1 g, sintinimab	
22nd August 2022	Adjuvant mitotane at 1 g, sintinimab	
20th September 2022	Sintinimab	
26th October 2022	Sintinimab	
26th November 2022	Sintinimab	
11th January 2023	Sintinimab	
8th February 2023	Sintinimab	
8th March 2023	Sintinimab	

EP, etoposide and paraplatin.

On 24th June 2021, the patient’s plasma aldosterone and cortisol decreased to 48 ng/L and 168 nmol/L, respectively (**Figure 1A**). He tolerated treatment except for the occurrence of endocrine adverse events. At the third cycle of a combination of EP-M and sintilimab, he developed hypoadrenocorticism and hypothyroidism, characterized by an elevated ACTH level of 522 pg/mL and an elevated TSH level of 5.953 mU/L (accompanied by FT3 level of 4.84 pmol/L and FT4 level of 11.36 pmol/L). Thus, a hormone replacement regimen (80 mg hydrocortisone and 50 μg l-thyroxine/day) was prescribed. Plasma cortisol levels maintained at 14.15–28.49 nmol/L, and TSH, FT3, and FT4 levels were normal throughout his subsequent treatment.

## Discussion

Here, we describe a patient with metastatic ACC who demonstrated long-term stability following cytoreductive surgery and a combination of EP-M and sintilimab.

Surgery is considered the most effective initial treatment for ACC, as evidenced by a previous study demonstrating reduced mortality in patients with metastatic ACC following cytoreductive surgery (7). In this case, the patient underwent palliative surgery to remove the primary lesion, and the pathological result confirmed the diagnosis of ACC.

Despite the patient’s highly aggressive pathology, with vascular invasion, a Ki-67% of 75%, and a mitotic count of 40/50 high-power field, the combination of chemotherapy with mitotane was selected as the initial regimen after surgery following the ESMO Guidelines. To avoid the cardiotoxic effect of doxorubicin, we modified the EDP chemotherapy regimen to EP, which was shown to have comparable efficacy with advanced ACC (8, 9). The patient’s response to treatment was initially disappointing, with metastatic lesions in the liver and lungs increasing in size after only 1 month.

Reports of the genetic analysis from tumor tissue and blood came out with informative data, showing that this patient had an MSI-H tumor, a splice mutation in DNA MMR machinery (MLH1), and a high TMB. Tumor MSI-H and/or MMR-deficient status is a known predictive biomarker of response to immune-based therapies (5, 10). Earlier observations demonstrated an increased response of ACC to the PD-1 inhibitor pembrolizumab in the presence of MSI-H or MMR deficiency (11). Hence, high TMB status is regarded as a potential biomarker for predicting the efficacy and response rate of immunotherapy (12, 13). After multidiscipline consultation of the above predictors of response to immunotherapy in ACC, we began to add sintilimab to the EP and mitotane regimen on 1 June 2021.

While immunotherapy is the latest evolution in ACC therapy, its efficacy varies widely (14). A phase 1b expansion cohort involving 50 metastatic ACC patients who received avelumab showed an objective response rate of 6.0% and a partial response (PR) in three patients over a median of 16.5 months (15). In a phase 2 clinical trial using pembrolizumab monotherapy in 16 metastatic ACC patients, two patients had PR, seven patients had SD, and five patients had PD, resulting in an objective response rate of 14% (16). In a small trial, only two out of 10 metastatic ACC patients had SD during the treatment with nivolumab (17). This evidence reveals that immunotherapy is effective for ACC with metastases in some patients. Recent evidence suggests that immunotherapy also provides clinically meaningful and durable antitumor activity even in patients with advanced ACC that is MSI-stable compared to those with MSI-H (18).

Sintilimab, a novel human IgG4 antibody targeting PD-1, binds 10–50-fold more strongly compared to pembrolizumab (19). Based on our institute’s experience, we selected sintilimab as the immunotherapy drug for this patient. We speculated that the exceptional effects of sintilimab were possibly associated with MSI-H and the high TMB observed in this patient, although there are different views on the use of immunotherapy for ACC. In addition, the combination of sintilimab and mitotane might be synergistic, as mitotane is the adrenolytic agent leading to steroid reduction, which can make the tumors more susceptible to checkpoint inhibitor therapy. Hypercortisolemia, whatever endogenous (due to tumor secretion) and exogenous glucocorticoid administrations, has been inversely associated with immune infiltration and has the potential to impair immunotherapy efficacy in ACC patients (20). Steroid hormone secretion was inversely associated with immune infiltration, so treatment with glucocorticoid inhibitor drugs may enhance the response to immunotherapy. Considering that high levels of glucocorticoids affect the effectiveness of immunotherapy, we administered mitotane to suppress the patient’s cortisol levels.

In conclusion, we report the first case of a stage IV ACC patient with metastatic lesions in both the lungs and liver who was treated with a combination of EP-M and sintilimab and has maintained long-term stable diseases. In summary, our patient’s unique gene mutations may be targets for amending treatment of advanced ACC to achieve longer survival with tolerable morbidity.

## Data availability statement

The original contributions presented in the study are included in the article/supplementary material. Further inquiries can be directed to the corresponding authors.

## Ethics statement

Written informed consent was obtained from the individual(s) for the publication of any potentially identifiable images or data included in this article.

## Author contributions

S-LZ conceived the idea of this manuscript. YW, X-YW, X-XF, Z-HL, and S-LZ clinically followed up on the patient. YW, LW, and Z-HL collected and interpreted the patient clinical data. YW wrote the manuscript. S-LZ revised the manuscript. LY and S-LZ were responsible for the supervision. All authors contributed to the article and approved the submitted version.
